# T250. CLINICAL CORRELATES OF SUBJECTIVE QUALITY OF LIFE IN INDIVIDUALS WITH AT-RISK MENTAL STATE FOR PSYCHOSIS IN HONG KONG

**DOI:** 10.1093/schbul/sby016.526

**Published:** 2018-04-01

**Authors:** Irving Teng, Wing Chung Chang, Hoi Ching Lee, Cheuk Fei Wong, Sui Fung Wo, Suet In Chan, Sanyin Chiu, Lai Ming Hui, Kit Wa Chan, Ho Ming Lee, Yi Nam Suen, Eric Chen

**Affiliations:** The University of Hong Kong

## Abstract

**Background:**

Subjective quality of life (SQoL) is an important outcome domain in individuals with at-risk mental state (ARMS) for psychosis. In an effort to better understand and maximize SQoL in ARMS populations, an increasing number of research has been conducted to investigate factors determining SQoL. This study aimed to examine clinical, functional and cognitive correlates of SQoL in Chinese young people presenting with ARMS in Hong Kong.

**Methods:**

This is a naturalistic prospective study examining the longitudinal course of ARMS and prediction of psychosis in Hong Kong. In total, 110 Chinese participants aged 15 to 40 years presenting with ARMS were recruited from a territory-wide specialized early intervention service for psychosis. ARMS status was verified using Comprehensive Assessment for At-Risk Mental State (CAARMS). Assessments encompassing symptom profiles (Positive and Negative Syndrome Scale, PANSS; Montgomery-Asberg Depression Rating Scale, MADRS; Brief Negative Symptom Scale, BNSS), functioning (Social and Occupational Functioning Rating Scale, SOFAS) and a brief battery of cognitive tests was conducted. A validated Chinese version of SF12 questionnaire was used to measure SQoL. The current analysis focused on data collected at baseline.

**Results:**

Of 110 ARMS participants, 48.2% were male. The mean age and educational level of the sample was 20.9 years (S.D.=6.7) and 11.4 years (S.D.=2.6), respectively. Correlation analyses revealed that SF12 mental health score was correlated with MADRS total score, BNSS total score and SOFAS score, while SF12 physical health score was correlated with PANSS positive symptom score only (p<0.05). Multiple linear regression analysis showed that only MADRS total score was independently associated with SF12 mental health score (p<0.001). SQoL measures were not correlated with any cognitive functions.

**Discussion:**

Our results were consistent with the literature which indicates that psychological domain of SQoL is significantly related to depressive symptoms in ARMS individuals. Further analysis on the longitudinal data regarding our prospective ARMS cohort will clarify variables predictive of SQoL at follow-up.

